# Detection of pathogenic microorganisms from bloodstream infection specimens using TaqMan array card technology

**DOI:** 10.1038/s41598-018-31200-3

**Published:** 2018-08-27

**Authors:** Chao Zhang, Xin Zheng, Chengna Zhao, Yan Li, Shuiping Chen, Gang Liu, Chengbin Wang, Qingyu Lv, Peng Liu, Yuling Zheng, Decong Kong, Hua Jiang, Yongqiang Jiang

**Affiliations:** 1 State Key Laboratory of Pathogen and Biosecurity, Academy of Military Medical Science, Beijing, China; 2 Anhui Medical University, Anhui, China; 3 The Affiliated Hospital Of Military Medical Science, Beijing, China; 4 Department of Infectious Disease, Beijing Children’s Hospital, Capital Medical University, Beijing, China; 5 Chinese People’s Liberation Army General Hospital (301 Hospital), Beijing, China

## Abstract

Bloodstream infections (BSIs) are often life-threatening, and rapid identification is critical. Here, we developed a TaqMan array card (TAC) assay to detect pathogens in BSI specimens. The TAC included 30 primer/probe pairs targeting 27 species and 3 controls. Reverse transcription and 0.1% blue dextran 2000 increased the TAC assay efficiency. The primer/probe pairs had a limit of detection of 10^0^–10^2^ CFU/mL and a specificity of 100%. For whole blood specimens, the TAC assay showed a sensitivity and specificity of 79.4% and 99.69%, respectively. For blood culture samples, the TAC assay showed a sensitivity and specificity of 100% and 99.67%, respectively. The TAC assay could be a promising method for early detection of bloodstream infection.

## Introduction

Severe infectious diseases such as sepsis cause serious morbidity and mortality^1^. Immunosuppressants, invasive diagnostic and therapeutic procedures, aging, and multidrug resistance development in pathogenic organisms could increase the incidence of bloodstream infections (BSIs). Timely antibiotic treatment is critical for a satisfactory prognosis of BSI. Studies have shown that when appropriate antimicrobial administration application was delayed, the chances of survival decreased by 7.6% per hour^2^. Effective management of sepsis relies on early accurate detection. However, the current gold standard diagnostic method for BSIs, a blood culture test, is time-consuming and has poor sensitivity for slow growing, intracellular and fastidious microorganisms and antimicrobial-treated patients. It usually takes a minimum of 48 hours to confirm a positive infection and 5 days to confirm a negative infection from a blood culture test^3,4^. Garnacho-Montero and colleagues reported that 12% of patients died from infection before microbiology culture test results became available^5^. The sensitivity of blood culture tests is approximately 60%, which is low and may cause patients to miss timely antibiotic treatments^6–9^. Because infections caused by different pathogens often show similar clinical presentation, patients are usually treated empirically when a precise pathogen diagnosis is not available. Thus, a rapid and accurate diagnostic method for BSIs will certainly improve patient care. In recent years, molecular diagnostic methods for BSIs have attracted considerable attention. Molecular diagnostic assays, such as polymerase chain reaction (PCR), can provide results within hours^3,4^. The LightCycler SeptiFast assay (Roche Molecular Diagnostics, Meylan, France) has now been authorized for whole blood specimen diagnosis in Europe. In addition, peptide nucleic acid fluorescent *in situ* hybridization (PNAFISH, AdvanDx, Woburn, USA) is FDA-approved for the diagnosis of enterococcal infections^2^. Although molecular microbial diagnosis of pathogens is useful and rapid, clinical evaluation showed that at the present stage, molecular diagnosis is used to complement blood culture rather than replace it.

Nucleic acid-based methods could shorten the turnaround times, and multiplex base PCR could simultaneously detect multiple pathogens. However, it also has its own limitations, such as the presence of PCR inhibitors and background DNA, low bacterial load, insufficient sensitivity and difficulty in establishing an assay capable of detecting a wide range of pathogens, especially when emergence of a new type or subtype is present. In addition, for some multiplex PCRs in the one-well system, if the components in the system are changed, the performance needs re-evaluation. As a result, the multiplex PCR-based method requires extensive optimization to be used in clinical settings.

The TaqMan array card (TAC) is a 384-well, singleplex qPCR-based system. The TAC technology is based on microfluidics, and the PCR volume in each well on a TAC is only 1–2 μL. Therefore, a TAC is particularly preferable for samples of limited quantity. In addition, a TAC allows for the analysis of 24–348 targets simultaneously^10^. TAC systems have previously been developed to diagnose bacterial, virus, and parasite-associated infections in respiratory diseases, enteric diseases, and neonatal sepsis^10–14^. However, for acute febrile illness case specimen detection, including whole blood and serum specimen, the limit of detection (LOD) is reported to be approximately 10^3^ CFU/ml. As a result, here, we aim to develop and optimize a TAC to detect pathogenic bacteria and fungi from BSI specimens, including blood culture specimens and whole blood specimens.

## Results

### Optimization for the TAC assay

**Figure 1:**
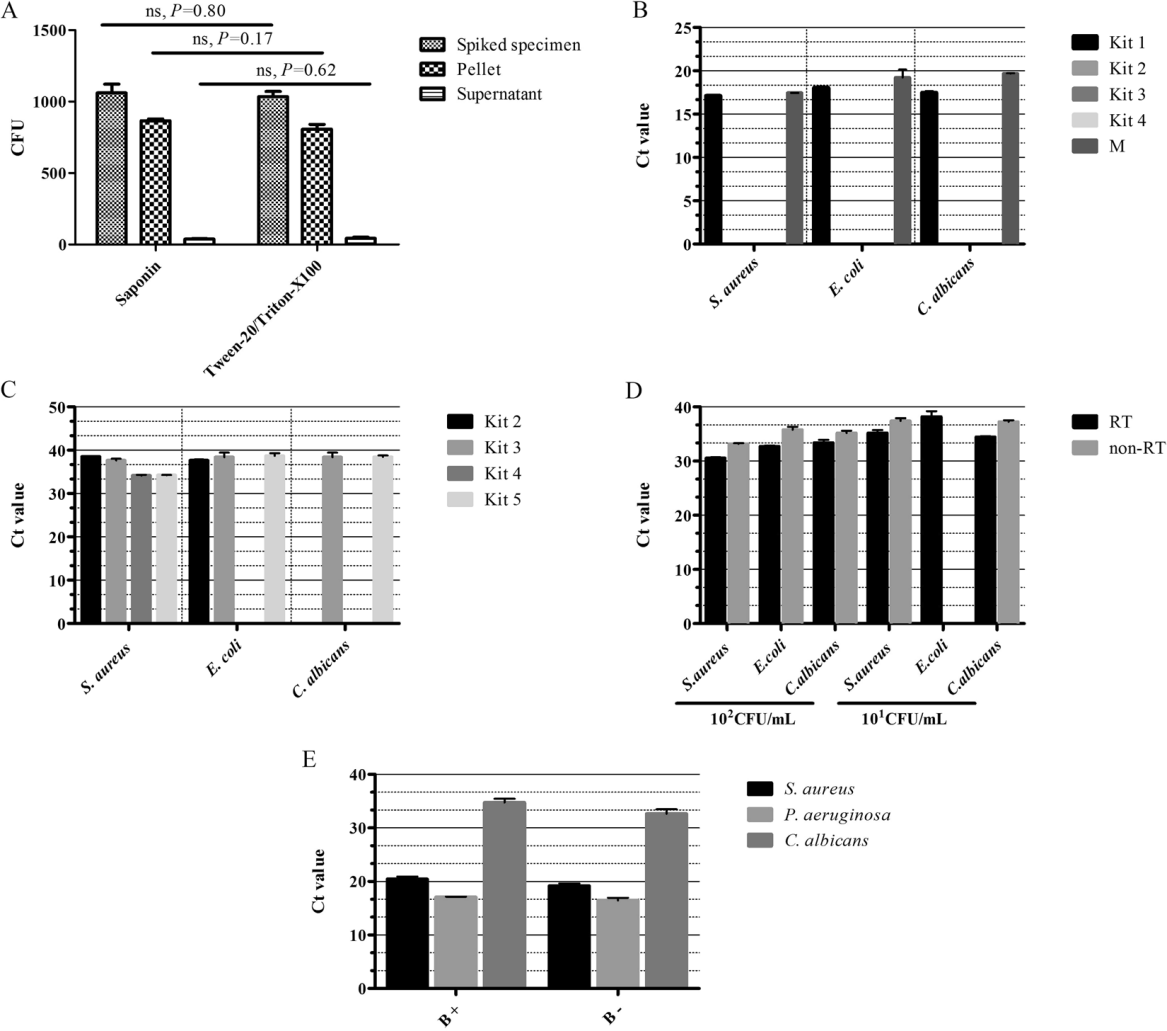
TAC optimization. (**A**) 50% Tween-20/Triton X-100 and 0.1% saponin treatment on the yield of pathogen cells from whole blood specimens. 10^3^ CFU *S. aureus* was mixed in 1 mL blood from healthy donors and then lysed by Tween-20/Triton X-100 or saponin. The colony numbers were determined by a plate count. (**B**,**C**). TNA extraction kit performance on blood culture specimens (**B**) and mock whole blood specimens (**C**). Kit 1 is the BiOstic bacteremia DNA isolation kit; Kit 2 is the QIAamp DNA Blood Mini Kit; Kit 3 is the QIAamp UCP Pathogen Mini Kit; Kit 4 is the TIANamp Blood DNA Kit; Kit 5 is the QIAamp cador Pathogen Mini Kit. M represents the benzyl alcohol-guanidine hydrochloride method. For blood culture specimens, three blood culture samples positive for *S. aureus* (G^+^), *E. coli* (G^−^), or *C. albicans* (fungi) were used. For whole blood specimens, three whole blood mock specimens spiked with 10^1^ CFU/mL of *S. aureus*, *E. coli*, and *C. albicans* were used. (**D**) The effect of TNA combined with reverse transcription on amplifying efficiency. 10^1^ and 10^2^ CFU *S. aureus* (G^+^), *E. coli* (G^−^), or *C. albicans* (fungi) were used to make mock specimens. The cycle threshold (Ct) was compared with RT or without RT. (**E**) The effect of 0.1% blue dextran 2000 on the TAC assay. 10^6^ CFU *S. aureus* (G^+^), 10^7^ CFU *P. aeruginosa* (G^−^) and 10^4^ CFU *C. albicans* (fungi) were used to make mock specimens. B+, TAC assay with blue dextran 2000; B−, TAC assay without blue dextran 2000.

To maximize pathogen cell yield from blood specimens, we used 50% Tween 20/Triton X-100 or 0.1% saponin to release pathogen cells engulfed by phagocytes^15,16^. Fifty percent Tween 20/Triton X-100 and 0.1% saponin resulted in similar yields of pathogen cells (Fig. 1A), and for easy preparation, 0.1% saponin was then used for total nucleic acid (TNA) extraction. To maximize bacterial or fungal TNA yield, we tested 4 TNA extraction kits and one method. Kits 1, 2, 3, and 4 and the benzyl alcohol-guanidine hydrochloride TNA extraction method were tested on blood culture samples showing positive results for *S. aureus* (G^+^), *E. coli* (G^−^), or *C. albicans* (fungi). The result indicated that when the TNA extracted by the benzyl alcohol-guanidine hydrochloride method and Kit 1 was used as template, a 1:10 dilution sufficiently removed matrix inhibition and resulted in an effective amplification. No amplification was observed for TNA when Kits 2, 3 and 4 were used as templates (Fig. 1B). Thus, Kit 1 (BiOstic bacteremia DNA isolation kit from MOBIO, Carlsbad, CA, USA) was chosen to extract TNA from blood culture samples instead of using benzyl alcohol, as it is toxic.

Kits 2, 3, 4 and 5 were tested on mock whole blood specimens. According to the epidemiological investigation, the representative bacteria *S. aureus* (G^+^), *E. coli* (G^−^), and *C. albicans* (fungus) were chosen to evaluate the optimized parameters in TAC. A healthy individual’s whole blood specimen was spiked with 10^1^ CFU/mL of *S. aureus*, *E. coli*, or *C. albicans*. For all three pathogens, only Kits 3 and 5 resulted in sufficient TNA extraction and amplification from the mock specimens (Fig. 1C). Kit 3 requires mechanical mincing pretreatment, which could potentially damage DNA and RNA integrity. Kit 5 uses an enzyme to lyse bacterial and fungal cell walls, which is gentler than mechanical mincing. Thus, Kit 5 (QIAamp Cador Pathogen Mini Kit from Qiagen, Hilden, Germany) was used to extract TNA from whole blood specimens for the subsequent TAC assay. To improve lysing efficiency, we used an enzyme cocktail composed of lysozyme (20 mg/mL), mutanolysin (300 U/mL), lysostaphin (40 U/mL), and lyticase (1000 U/mL).

Because the concentration of bacteria or fungi in whole blood specimens is usually very low, extraction of both DNA and RNA in combination with subsequent reverse transcription (RT) might improve detection efficiency. We then compared the cycle threshold (Ct) value decrease by using TNA directly as templates versus using TNA + RT as templates. When the TNA extracted from mock specimens spiked with 10^1^–10^2^ CFU/mL *S. aureus*, *E. coli*, or *C. albicans* was used directly as templates without RT, the Ct value was 1 to 3 times higher than the Ct value when TNA + RT was used as a template (Fig. 1D). These data suggest that using TNA + RT may improve TAC performance.

To ensure an even distribution of TNA templates on the TAC card, 0.1% blue dextran 2000 was added in TNA templates according to previous studies^17,18^. We tested the effects of 0.1% blue dextran 2000 on the TAC assay using mock specimens spiked with *S. aureus*, *P. aeruginosa*, or *C. albicans*. The Ct value was reduced by 1 to 2 when 0.1% blue dextran 2000 was used (Fig. 1E). These results indicate that 0.1% blue dextran 2000 may improve TNA template distribution on the TAC card. The results indicate that with all the optimization procedures described above, the LOD on TAC could reach ≤10^2^ CFU/mL.

### Evaluation of primer/probe on TAC

**Figure 2:**
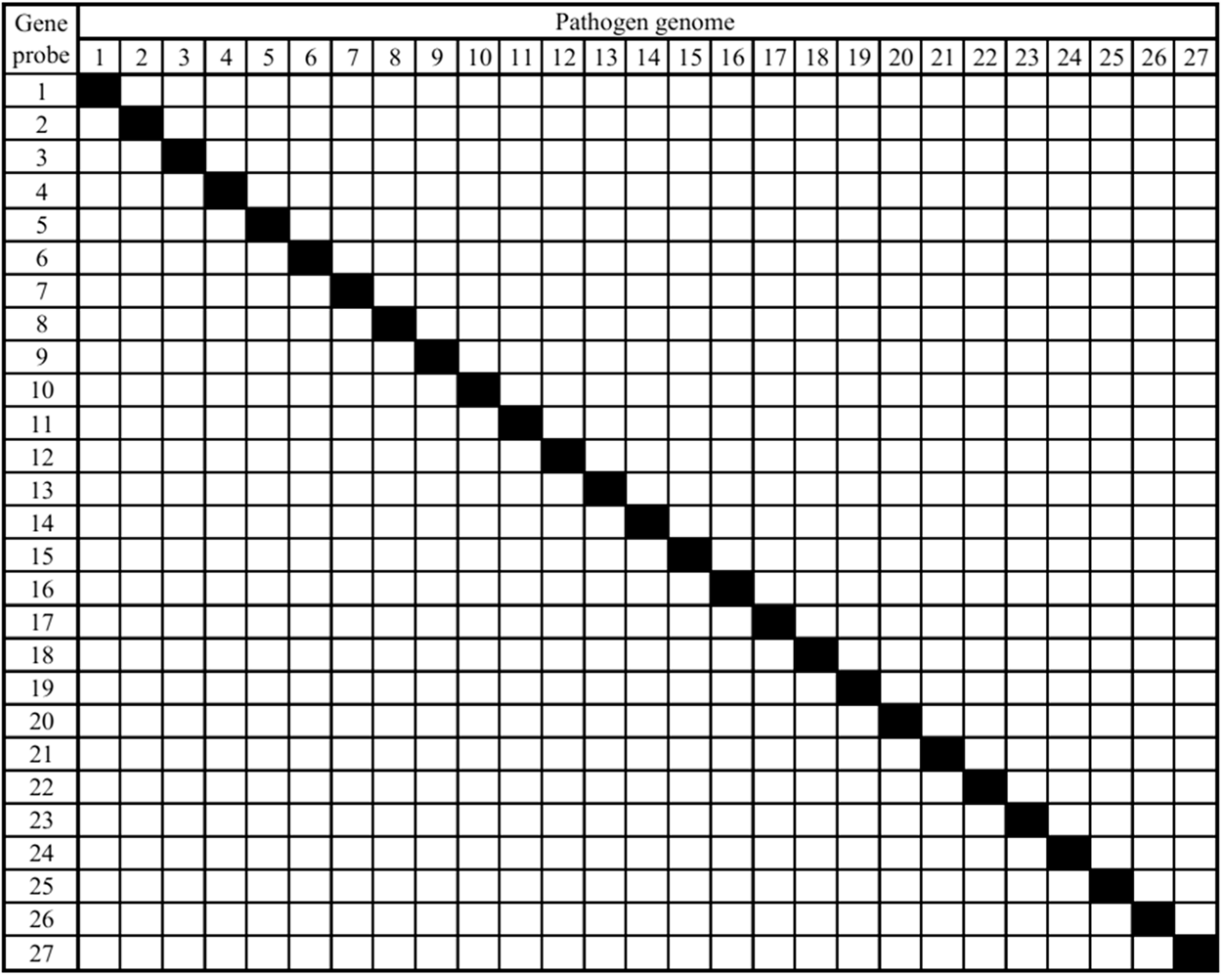
Specificity of primer and probe pairs evaluation. The specificity of primers and probes was evaluated using mock specimens spiked with 1–27 bacterial culture isolates at a concentration of 10^5^ CFU/mL. The rows represent primers and probes, and columns represent TNA extracted from the mock specimens spiked with each bacterial culture. The keys to the numbers are as follows: 1: *S. aureus*; 2: *S. epidermidis*; 3: *S. hominis*; 4: *S. haemolyticus;* 5: *K. oxytoca*; 6: *E. faecium*; 7: *E. faecalis*; 8: *S. pneumoniae*; 9: *S. agalactiae*; 10: *S. pyogenes*; 11: *C. perfringens*; 12: *S. marcescens*; 13: *L. monocytogenes*; 14: *E. coli*; 15: *K. pneumoniae*; 16: *Ab*; 17: *S. maltophilia*; 18: *P. aeruginosa*; 19: *A. xylosoxidans*; 20: *B. cepacia*; 21: *N. meningitides*; 22: *H. influenzae*; 23: *C. neoformans*; 24: *C. glabrata*; 25: *C. tropicalis*; 26: *C. albicans*; 27: *C. parapsilosis*.

Mock specimens spiked with 27 targeted pathogens were prepared to determine the LOD, specificity, and amplification reproducibility of the primer/probe. The LOD was ≤10^2^ CFU/mL (Table 1), and the specificity for all was 100% (Fig. 2). These results suggested that the primers and probes appear to be valid for a TAC assay. The R^2^ value was ≥0.99 for most of the primer and probe pairs, except *S. haemolyticus* (R^2^ = 0.970), *E. faecium* (R^2^ = 0.924), *K. oxytoca* (R^2^ = 0.985) and *Ab* (R^2^ = 0.979). Nevertheless, the five primer and probe pairs showed a very low LOD (10^0^–10^1^ CFU/mL, Table 1). The coefficients of variation (CVs) of intra-assay and inter-assay were all <5% and <12%, respectively (Table 1). These results indicate that the 27 pairs of probes and primers may be specific, sensitive, and associated with high amplification reproducibility.

**Table 1:** LOD and amplification reproducibility of the primer and probe on TAC.

Targeted pathogens	Linearity R^2^ efficiency (%)	Limit of detection (LOD) (CFU/mL)	Ct at LOD	Reproducibility of amplification efficiency CV	
				Inter-assay	Intra-assay
*S. aureus*	0.999 (91.136)	10^1^	35.94	1.48%	0.28%
*S. epidermidis*	1.000 (93.478)	10^1^	38.48	0.73%	1.69%
*S. hominis*	0.998 (90.83)	10^0^	39.08	3.37%	6.33%
*S. haemolyticus*	0.970 (95.199)	10^0^	35.11	1.99%	9.63%
*K. oxytoca*	0.985 (85.925)	10^1^	37.51	1.47%	3.08%
*E. faecalis*	1.000 (95.176)	10^1^	37.96	1.22%	1.45%
*E. faecium*	0.924 (98.241)	10^1^	35.75	1.20%	5.00%
*S. pneumoniae*	0.999 (92.845)	10^1^	34.59	1.47%	0.83%
*S. agalactiae*	0.999 (93.03)	10^1^	37.91	0.60%	8.20%
*S. pyogenes*	0.992 (95.359)	10^0^	36.80	1.31%	11.58%
*C. perfringens*	0.992 (89.379)	10^2^	37.85	0.46%	1.58%
*S. marcescens*	0.996 (96.078)	10^1^	38.19	1.84%	5.66%
*L. monocytogenes*	0.999 (102.677)	10^1^	36.06	1.90%	2.75%
*E. coli*	0.993 (102.611)	10^1^	36.29	2.74%	7.50%
*K. pneumoniae*	1.000 (91.873)	10^1^	37.94	0.94%	0.40%
*Ab*	0.979 (102.292)	10^1^	37.11	0.38%	6.37%
*S. maltophilia*	0.996 (91.848)	10^0^	37.89	1.58%	2.08%
*P. aeruginosa*	0.992 (92.918)	10^2^	36.28	1.31%	1.68%
*A. xylosoxidans*	0.991 (100.652)	10^0^	38.04	1.33%	0.35%
*B. cepacia*	0.999 (90.976)	10^1^	38.14	1.88%	2.65%
*N. meningitides*	0.998 (95.360)	10^2^	36.92	0.40%	0.72%
*H. influenzae*	0.999 (92.914)	10^1^	36.42	0.67%	1.70%
*C. neoformans*	0.996 (93.441)	10^1^	38.26	1.30%	2.49%
*C. glabrata*	0.999 (92.075)	10^1^	37.72	1.17%	1.52%
*C. tropicalis*	0.996 (96.290)	10^1^	38.99	0.41%	7.62%
*C. albicans*	0.995 (91.746)	10^1^	34.90	4.49%	3.74%
*C. parapsilosis*	0.999 (93.193)	10^0^	34.17	0.59%	0.76%

In addition, 7 pathogens, which have been found in BSIs^11^ but not included in TAC, were also used to evaluate the specificity of TAC. The 7 pathogens are *Coxiella burnetii*, *Rickettsia prowazekii*, herpes simplex virus, tick-borne virus, Japanese encephalitis virus, dengue virus, and *Aspergillus nidulans*. The genomic DNA or RNA of the 7 pathogens showed no cross-reactions on the TAC card, which also confirmed the specificity.

### TAC performance on blood culture specimens

We next validated the TAC card using 151 blood culture samples. According to the bacterial culture test results, 129 were positive for the targeted pathogens on the TAC card, and 22 were culture-negative specimens. The TAC assay results of the 129 samples were consistent with the bacterial culture test results, and thus, the sensitivity of the TAC assay for blood culture samples was 100% (Table 2). The specificity of the TAC assay for different targeted pathogens ranged from 97% to 100% (Table 2). Notably, in the negative specimens, 13 targets were identified by the TAC assay (Table 2), and the results were confirmed by PCR/sequencing.

**Table 2:** Validation of the TAC with the blood culture samples.

Target	TAC	Blood culture test		TAC sensitivity (%)	TAC specificity (%)
		Positive	Negative		
*S. aureus*	Positive	9	2	100.0%	98.6%
	Negative	0	140		
*S. epidermidis*	Positive	22	1	100.0%	99.2%
	Negative	0	128		
*S. hominis*	Positive	6	4	100.0%	97.2%
	Negative	0	141		
*S. haemolyticus*	Positive	18	2	100.0%	98.5%
	Negative	0	131		
*E. faecium*	Positive	10	2	100.0%	98.6%
	Negative	0	139		
*E. faecalis*	Positive	1	0	100.0%	100.0%
	Negative	0	150		
*S. pneumoniae*	Positive	9	0	100.0%	100.0%
	Negative	0	142		
*S. agalactiae*	Positive	1	0	100.0%	100.0%
	Negative	0	150		
*E. coli*	Positive	26	0	100.0%	100.0%
	Negative	0	125		
*K. pneumoniae*	Positive	3	2	100.0%	98.6%
	Negative	0	146		
*Ab*	Positive	21	0	100.0%	100.0%
	Negative	0	130		
*P. aeruginosa*	Positive	2	0	100.0%	100.0%
	Negative	0	149		
*C. albicans*	Positive	1	0	100.0%	100.0%
	Negative	0	150		

### Testing the TAC card on whole blood specimens

We then used 183 whole blood specimens to test the TAC card. Of the whole blood specimens, 102 were positive for the targeted pathogens on the TAC card, and 81 showed negative results from the bacterial culture tests. The bacterial culture test results were obtained from the diagnostic laboratory of the hospital. The TAC assay revealed that 81 of the 102 positive whole blood specimens were positive, and thus, the overall sensitivity of the TAC assay was 79.4%. The sensitivity of the TAC assay for each individual targeted pathogen ranged from 50% to 100% (Table 3). The average specificity was 99.69%, and the specificity of the TAC assay for each individual targeted pathogen ranged from 98.4% to 100% (Table 3). Although the 81 samples displayed negative results for the targeted pathogens from the bacterial culture tests, the patient did present with clinical symptoms of infection. Here, of the 81 negative whole blood specimens, 15 specimens were identified by the TAC assay to be positive for the targeted pathogens on the TAC, and the results of the TAC assay were confirmed by PCR/sequencing (Table 3).

**Table 3:** Testing the TAC using the whole blood specimens.

Target	TAC	Blood culture test		TAC		PPV	NPV
		Positive	Negative	Sensitivity (%)	Specificity (%)		
*S. aureus*	Positive	7	1	87.5%	99.4%	87.5%	99.4%
	Negative	1	174				
*S. epidermidis*	Positive	11	2	78.6%	98.8%	84.6%	98.2%
	Negative	3	167				
*S. hominis*	Positive	8	1	80.0%	99.4%	88.9%	98.8%
	Negative	2	172				
*S. haemolyticus*	Positive	1	0	100.0%	100.0%	100.0%	100.0%
	Negative	0	182				
*E. faecium*	Positive	4	2	100.0%	98.9%	66.7%	100.0%
	Negative	0	177				
*E. faecalis*	Positive	2	1	66.7%	99.4%	66.7%	99.4%
	Negative	1	179				
*S. pneumoniae*	Positive	10	1	83.3%	99.4%	90.9%	98.8%
	Negative	2	170				
*S. agalactiae*	Positive	1	0	50.0%	100.0%	100.0%	99.4%
	Negative	1	181				
*E. coli*	Positive	17	1	70.8%	99.4%	94.4%	95.8%
	Negative	7	158				
*K. pneumoniae*	Positive	7	2	70.0%	98.8%	77.8%	98.3%
	Negative	3	171				
*Ab*	Positive	12	1	92.3%	99.4%	92.3%	99.4%
	Negative	1	169				
*P. aeruginosa*	Positive	1	3	100.0%	98.4%	25.0%	100.0%
	Negative	0	179				

PPV, positive predictive value; NPV, negative predictive value.

### Comparison of TAC and blood culture

Of the whole blood specimens, 21 were culture positive/TAC negative. To explain the discrepancy, specimen data were analyzed. Five specimens were collected after antibiotic treatment. Nine specimens were stored more than 2 years, and 7 specimens were stored as blood clots (Table 4). Antibiotic treatment might have resulted in dead pathogens in the blood culture specimen. Studies have reported that longtime storage of whole blood samples might cause nucleic acid degradation, which may contribute to the failure of the TAC assay^11^. For the TAC assay, it is not suitable to detect pathogens in blood clots and serum^11^. These reasons might contribute to the inconsistencies described above. For the 15 whole blood specimens and 13 blood culture specimens, which were culture negative/TAC positive, the results were all confirmed by PCR/sequencing. In addition, the results were the same as those of TAC (Table 5). To verify the specificity of TAC, 49 whole blood specimens from healthy donors were evaluated, and no amplification was observed.

**Table 4:** Comparison between TAC and culture method on whole blood specimens.

Blood culture	Number	Antibiotic treatment	Storage time	Blood clot	TAC result	PCR/sequencing
*S. aureus*	1	—	—	+	Negative	Negative
*S. hominis*	3	—	—	+	Negative	Negative
*S. epidermidis*	2	—	>2 years	−	Negative	Negative
*E. faecalis*	1	—	—	+	Negative	Negative
*S. pneumoniae*	2	After antibiotic therapies	—	−	Negative	Negative
*S. agalactiae*	1	After antibiotic therapies	—	−	Negative	Negative
*E. coli*	2	—	—	+	Negative	Negative
	4	—	>2 years	−	Negative	Negative
	1	After antibiotic therapies	—	−	Negative	Negative
*K. pneumoniae*	3	—	>2 years	−	Negative	Negative
*Ab*	1	After antibiotic therapies	—	−	Negative	Negative
Negative	1	—	—	−	*S. aureus*	*S. aureus*
Negative	2	—	—	−	*S. epidermidis*	*S. epidermidis*
Negative	1	—	—	−	*S. hominis*	*S. hominis*
Negative	2	—	—	−	*E. faecium*	*E. faecium*
Negative	1	—	—	−	*E. faecalis*	*E. faecalis*
Negative	1	—	—	−	*S. pneumoniae*	*S. pneumoniae*
Negative	1	—	—	−	*E. coli*	*E. coli*
Negative	2	—	—	−	*K. pneumoniae*	*K. pneumoniae*
Negative	1	—	—	−	*Ab*	*Ab*
Negative	3	—	—	−	*P. aeruginosa*	*P. aeruginosa*

**Table 5:** Comparison between TAC and culture method on blood culture specimens.

Blood culture result	TAC result	Number of discrepancies	PCR/sequencing
Negative	*S. aureus*	2	*S. aureus*
Negative	*S. epidermidis*	1	*S. epidermidis*
Negative	*S. hominis*	4	*S. hominis*
Negative	*S. haemolyticus*	2	*S. haemolyticus*
Negative	*E. faecium*	2	*E. faecium*
Negative	*K. pneumoniae*	2	*K. pneumoniae*

## Discussion

TAC technology appears to be a useful tool for identifying pathogenic organisms from clinical specimens efficiently and effectively. A TAC assay is a relatively closed system, which could avoid potential contamination from the environment more effectively than with PCR-based methods. Another benefit of a TAC is that as a singleplex system, assays in TAC are independent of each other. Therefore, changing one primer/probe pair does not affect the system too much. In addition, it gives the end users the ability to prepare his or her own plate. However, as a small reaction system, without sufficient optimization, it may not meet the demand for clinical detection.

In the current study, parameters that focused on the amplification of the template concentration and on the template distribution were fully optimized and evaluated in a TAC system to detect pathogenic bacteria and fungi from clinical BSI specimens. The number of pathogen cells in whole blood specimens is often very low, in most cases approximately 10^0^–10^1^ CFU/mL^19^. Accordingly, for clinical whole blood specimen detection, obtaining enough template is a challenge for TAC detection. To extract sufficient pathogen TNA from blood specimens, we used whole blood but not plasma or serum because white blood cells in whole blood, such as macrophages, may contain engulfed pathogens^7,16^. However, when whole blood specimens are used, the presence of a large amount of human genomic DNA after cell disruption could interfere with pathogen detection by PCR. A previous study has shown that in TNA extracted from whole blood samples, the human genomic DNA concentration is 20–60 µg/mL, whereas that of pathogen DNA is only 50 fg/mL–50 pg/mL^15^. To minimize such interference, it is reported that targeted enrichment is preferable^20^. In the current study, we aimed to enrich TNA from all pathogens in whole blood samples, and thus, targeted enrichment may not be applicable in our study. To eliminate the potential interference of human genomic DNA in the TAC assay, we used the surfactant 0.1% saponin to release pathogen cells from blood cells and then chose the preferred kit to extract pathogen TNA.

Blood culture samples contain sodium polyanetholesulfonate, which stays in the TNA extract and inhibits PCR and thus could interfere in the TAC assay^21^. In our preliminary experiments, we tried to remove the inhibitor by using a kit or benzyl alcohol-guanidine hydrochloride, but neither was effective. Ultimately, we chose the BiOstic bacteremia DNA isolation kit, which was suitable on a TAC on culture specimens at 10-fold serial dilution.

In addition to optimizing TNA extraction by selecting the TNA extraction kit showing the best performance, we also used blue dextran 2000 to enhance the even distribution of template on the TAC. Blue dextran 2000 has been used previously in digital PCR assays^17^, and in TAC assays, blue dextran 2000 was mixed with TNA to facilitate nucleic acid distribution.

For 28 culture-negative/TAC-positive specimens, our TAC assay revealed that they contained the targeted pathogens. In addition, PCR/sequencing confirmed the result. Inappropriate culture broth, the presence of dead pathogens because of antibiotic treatment, or inappropriate timing of blood collection could also contribute to the failure of the bacterial culture test to identify the pathogens^22^. For the 21 culture-positive/TAC-negative whole blood specimens, the quality of the samples, such as storage time, coagulation state, and blood collection time, had a considerable effect on the results.

Currently, there are some commercial diagnostic tests based on multiplex real-time PCR used in clinical specimen detection, such as the LightCycler SeptiFast assay (Roche Molecular Diagnostics, Meylan, France), which is used in blood pathogen detection in severe sepsis and septic shock^23,24^; Verigene BC-GP/GN (Nanosphere, Northbrook, USA) and Magicplex (Seegene, Seoul, Korea) in antimicrobial stewardship programs^25,26^; and BioFire FilmArray (BioFire Diagnostics, Salt Lake City, USA) in decreased infection control isolation time^3,27^. These tests have been compared with the blood culture test on both positive blood culture samples and whole blood specimens. Meta-analyses have indicated that all of the multiplex PCR assays show better performance on blood culture specimens, and the sensitivity and specificity of a multiplex PCR are 80.4–100% and 93–100%, respectively^3,4^. Our optimized TAC assay showed a sensitivity of 100% and a specificity of 97.2–100%. These results suggest that the optimized TAC assay appears to be effective when used to detect pathogens from blood culture samples. For whole blood specimens, the LOD of some multiplex PCR tests can be as low as 3–30 CFU/mL, but the sensitivity and specificity varied greatly from 37 to 95% and from 77 to 100%, respectively^3,4^. Here, our optimized TAC assay showed a sensitivity of 50–100% and a specificity of 98.4–100%.

## Conclusion

In this study, after primer/probe design and evaluation, we developed a TAC assay to simultaneously detect 27 bacterial and fungal species. By fully optimizing the performance of this card was improved, with an LOD of 10^0^–10^2^ CFU/mL and a specificity of more than 97% on both culture specimens and whole blood specimens within less than 4 hours. TAC itself is an excellent technique that avoids many shortcomings of PCR-based molecular detection methods. By fully optimizing its use, here, the TAC assay progressed one step closer to its future clinical application and will be a promising method for early identification of pathogens in BSI specimens.

## Materials and Methods

### Ethical statement

For experiments involving human blood samples, signed informed consent was obtained from all the patients or their guardians and healthy volunteers. All the protocols for handling patients’ or healthy donors’ blood specimens were approved by the Institutional Medical Ethics Committee of the Academy of Military Medical Sciences (AMMS). All methods performed in the study were in accordance with the ethical standards of the institutional and/or national research committee and with the 1964 Helsinki Declaration and its later amendments or comparable ethical standards.

### TAC design

**Figure 3:**
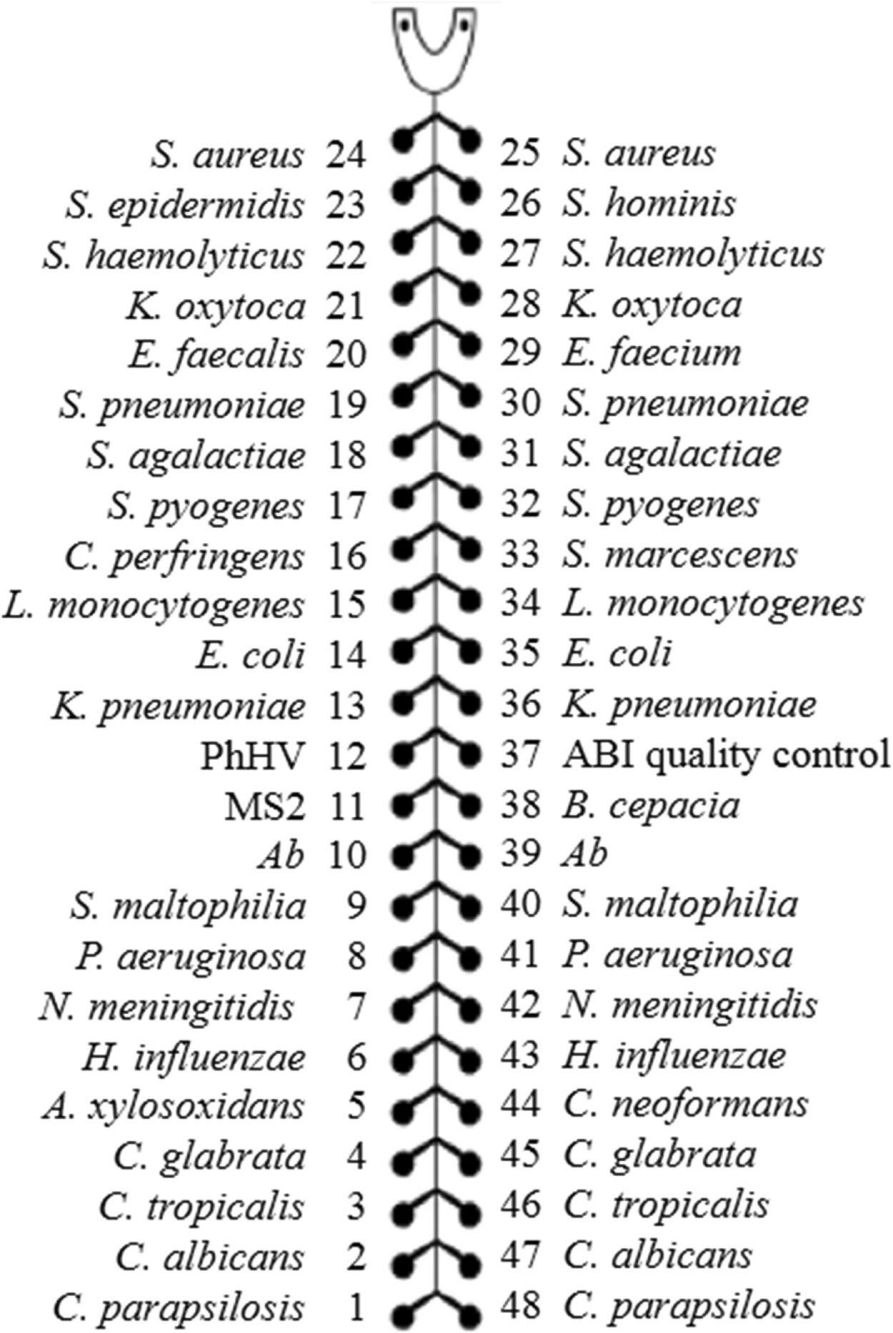
Primer and probe layout on the TAC card. A total of 27 primer/probe pairs were printed on the TAC. MS2 and PhHV were used as internal positive controls. Human 18S rRNA (ABI quality control) was used as a quality control for the TAC card. The 27 primer/probe pairs included *Staphylococcus aureus* (*S. aureus*)*, Staphylococcus epidermidis* (*S. epidermidis*), *Staphylococcus hominis* (*S. hominis*)*, Staphylococcus haemolyticus* (*S. haemolyticus*), *Klebsiella oxytoca* (*K. oxytoca*), *Enterococcus faecium* (*E. faecium*), *Enterococcus faecalis* (*E. faecalis*), *Streptococcus pneumoniae* (*S. pneumoniae*), *Streptococcus agalactiae* (*S. agalactiae*), *Streptococcus pyogenes* (*S. pyogenes*), *Clostridium perfringens* (*C. perfringens*), *Serratia marcescens* (*S. marcescens*), *Listeria monocytogenes* (*L. monocytogenes*), *Escherichia coli* (*E. coli*), *Klebsiella pneumoniae* (*K. pneumoniae*), *Acinetobacter baumannii* (*Ab*), *Stenotrophomonas maltophilia* (*S. maltophilia*), *Pseudomonas aeruginosa (P. aeruginosa), Achromobacter xylosoxidans (A. xylosoxidans), Burkholderia cepacia (B. cepacia), Neisseria meningitides (N. meningitides), Haemophilus influenza (H. influenzae), Cryptococcus neoformans (C. neoformans),Candida glabrata (C. glabrata), Candida tropicalis (C. tropicalis), Candida albicans (C. albicans), Candida parapsilosis (C. parapsilosis)*.

A total of 27 species-specific primer/probe pairs were printed on a TAC, including 11 Gram-positive bacteria (*Staphylococcus aureus*, *Staphylococcus hominis*, *Staphylococcus haemolyticus*, *Enterococcus faecalis*, *Streptococcus pneumoniae*, *Streptococcus agalactiae*, *Clostridium perfringens*, *Staphylococcus epidermidis, Enterococcus faecium, Streptococcus pyogenes*, and *Listeria monocytogenes*), 11 Gram-negative bacteria (*Klebsiella pneumoniae*, *Serratia marcescens, Acinetobacter baumannii*, *Stenotrophomonas maltophilia*, *Achromobacter xylosoxidans, Burkholderia cepacia, Escherichia coli, Klebsiella oxytoca, Pseudomonas aeruginosa, Haemophilus influenza, Neisseria meningitides, Clostridium perfringens)*, 5 fungal isolates (*Candida albicans*, *Candida glabrata*, *Candida parapsilosis*, *Cryptococcus neoformans, and Candida tropicalis*), 2 pairs of internal positive controls (MS2 for RNA and PhHV for DNA) and one pair specific to human 18S rRNA (ABI quality control) as a TAC quality control (Fig. 3). The TAC was manufactured by Life Technologies (Carlsbad, CA,USA).

### Primer/probe design

Oligonucleotide sequences of primers and probes were designed using Primer Express 3.0 software (Applied Biosystems, Foster City, CA, USA). The melting temperature (Tm) was optimized and intra- and inter-molecular interactions minimized when primers and probes were designed. Clustal X software (Life Technologies, Carlsbad, CA, USA) was used for sequence alignment analysis. The specificity and consensus of primer and probe sequences were also examined in the NCBI database using the BLAST tool.

The primer and probe sequences specific to *S. hominis*, *S. haemolyticus*, *K. oxytoca*, *S. maltophilia*, *A. xylosoxidans*, *B. cepacia*, and *C. neoformans* were not available from previous publications and thus were designed as a part of this study. For the remaining targeted pathogens on the TAC, the primer and probe sequences were obtained from previous publications and adapted to meet the universal cycling conditions. Therefore, 30 pairs of primers and probes were printed on the TAC. Primers and probes are listed in Table 6.

**Table 6:** Primer and probe sequences.

Pathogen	Target gene	Target size (bp)	Sequences (5′-3′)	Pathogen	Target gene	Target size (bp)	Sequences (5′-3′)
**Bacteria**							
S. aureus* ^30^	*Glutamate synthase*	130	F: ACGGTTAGGTGAATTGATTGTTTTAT	S. epidermidis* ^31^	*tpi*	128	F: CATCTGATAAACCTTCGACAGCTTT
			R: CGCATTTGAGCTGAAGTTG				R: TGCTATCTTCAATCACGGTATGAC
			P: TAKGACCAYCACGRGTC				P: TTCACGTTCTTCATCAGATT
*S. hominis* ^*#*^	*16s*	91	F: GGTTCGATAGTGAAAGATGGCT	*S. haemolyticus* ^*#*^	*sodA*	132	F: CAGTTGAGGGAACAGATCTTGAA
			R: GTTGCCTTGGTAAGCCGTT				R: CCCAGAATAATGAGTGATTTAAGTGTC
			P: AGATGGACCTGCGCCGTA				P: TCAAACAGCTGTTCGTAATA
*K. oxytoca* ^*#*^	*bla*	215	F: ATGCGCTGGGCGAACA	E. faecium* ^32^	*SodA*	138	F: GGGAAATCATGGCACCAAAT
			R: CTGCTGCGGCTGGGTAA				R: CCCATCCAGAACCAAATCGT
			P: AAGGCAATACCACCGGC				P: TGGTGGCGAACCTA
E. faecalis* ^32^	*recN*	73	F: GTATCGCGCACTCGAAGCC	S. pneumoniae* ^33^	*lytA*	75	F: ACGCAATCTAGCAGATGAAGCA
			R: CATGTCCATTCTTTGGGCAA				R: TCGTGCGTTTTAATTCCAGCT
			P: AGTCAGAAAGCGACAAAA				P: AACGCTTGATACAGGGAG
S. agalactiae* ^34^	*cfb*	150	F: TTCACCAGCTGTATTAGAAGTACATGC	S. pyogenes* ^35^	*spy*	98	F: GCACTCGCTACTATTTCTTACCTCAA
			R: CCCTGAACATTATCTTTGATATTTCTCA				R: GTCACAATGTCTTGGAAACCAGTAAT
			P: CAAGCCCAGCAAATGGCTC				P: AACTCATCAAGGATTTCT
C. perfringens* ^36^	*plc*	85	F: AAAAGAAAGATTTGTAAGGCGCTTAT	S. marcescens* ^37^	*luxS*	171	F: CTGGAAAGCGGCGATGG
			R: CCCAAGCGTAGACTTTAGTTGATG				R: GCCAGCTCGTCGTTGTGGT
			P: TGCCGCGCTAGCA				P: CACATGCACTCGCTG
L. monocytogenes* ^38^	*hly*	64	F: CATGGCACCACCAGCATCT	E. coli* ^39^	*uidA*	107	F: AGAACGGTTTGTGGTTRATCAGGA
			R: ATCCGCGTGTTTCTTTTCGA				R: CGTCACAGCCAAAAGCCAG
			P: CTTAGGACTTGCAGGCG				P: ACTGACCGGATRCCGA
K. pneumoniae* ^30^	*Diguanylatecyclase*	133	F: TGCAGATAATTCACGCCCAG	Ab* ^40^	*ompA*	92	F: AGTTCTTGGTGGTCACTTGAAGC
			R: ACCCGCTGGACGCCAT				R: TTAACTCTTGTGGTTGTGGAGCA
			P: CGCTCATCTGTTTCGC				P: AGTTGAACCAACTCCA
*S. maltophilia* ^*#*^	*23S*	181	F: GAACGCGGACTAGCCCTTAAG	P. aeruginosa* ^41^	*gyrB*	187	F: GGCGTGGGTGTGGAAGTC
			R: TCGGTCAGTAGTATTTAGCCTTGGA				R: TGGTGGCGATCTTGAACTTYTT
			P: TAGAAGGTGATAGCCCTGTAT				P: TGCAGTGGAACGACA
*A. xylosoxidans* ^*#*^	*nosZ*	166	F: AGCCAGATGGTCAAGTGGAACA	*B. cepaci* ^#^	*recA*	180	F: GGCGCCTGGTACAGCTACAA
			R: TGTTCAGCGACACCAGCCA				R: CTCTTCTTCGTCCATCGCCTC
			P: TCGACCCGATCATC				P: AACGCGCGTGAATT
N. meningitides*^38^	*ctrA*	114	F: TGTGTTCCGCTATACGCCATT	H. influenzae*^38^	*bexA*	116	F: GGACAAACATCACAAGCGGTTA
			R: GCCATATTCACACGATATACC				R: TGCGGTAGTGTTAGAAAATGGTATTATG
			P: AGAACGTCAGGATAAAT				P: TTGTAGTATTGATACGCTTTGT
**Fungi**							
*C. neoformans* ^*#*^	*CAP10*	183	F: CATTTGGGAGATGGGGATATAGTG	C. glabrata* ^42^	*26s*	124	F: GCGCCCCTTGCCTCTC
			R: GTTACTCTGCCCGTCTCTTTGCT				R: CCCAGGGCTATAACACTCTACACC
			P: CTGGAGCCATTCGG				P: TGGGCTTGGGACTCT
C. tropicalis*^42^	*28s*	117	F: GCGGTAGGAGAATTGCGTT	C. albicans* ^42^	*26s*	128	F: CTTGGTATTTTGCATGYTGCTCTC
			R: TCATTATGCCAACATCCTAGGTATA				R: GTCAGAGGCTATAACACACAGCAG
			P: CTCAGTCTAGGCTGGCAT				P: TGCGTTTACCGGGCCA
C. parapsilosis* ^42^	*28s*	107	F: GATCAGACTTGGTATTTTGTATGTTACTCT				
			R: CAGAGCCACATTT CTTTGCA				
			P: AGTTTACCGGGCCAGCA				

FAM and NFQ-MGB are the fluorophores labeled on probe. *Data are presented asRepresents modified. ^#^Represents designed *de novo* in this study.

### Specimen collection

Three types of samples were used in this study. (1) Mock (spiked) whole blood specimens were prepared as previously described^28^. Briefly, the absence of the spiked pathogen in healthy individuals’ whole blood specimens was first confirmed by a PCR/sequencing test. Then, the 27 bacteria were cultured and were diluted in 1 mL of the healthy individuals’ whole blood specimens. The taxonomic identity of the bacterial and fungal strains was confirmed by morphological examination, mass spectrometry (MS), and sequencing analysis. The bacteria were cultured in broth until reaching the mid-exponential growth phase, and colony numbers were then determined by a plate count. (2) Whole blood specimens from patients with febrile illness were collected from the 307^th^ Hospital of the Chinese People’s Liberation Army, Department of Infectious Disease of Beijing Children’s Hospital and Chinese People’s Liberation Army General Hospital (301 Hospital). EDTA was used as the anticoagulant when the blood specimens were collected. The blood specimens were stored at 4 °C for short-term storage or −70 °C for long-term storage. All of the 183 whole blood samples had been tested by bacterial culture. (3) A total of 151 blood culture samples were also obtained from the 307^th^ Hospital of the Chinese People’s Liberation Army and Department of Infectious Disease of Beijing Children’s Hospital. A minimum of 2 mL of blood specimens or blood culture samples was used for nucleic acid extraction. The specimens were stored at −70 °C.

### TAC optimization

Briefly, 0.1% saponin (Sigma-Aldrich, Saint Louis, USA) or 50% Tween-20/Triton X-100 (2% Tween-20 mixed with 1% Triton X-100 in equal volume) was first added in mock whole blood specimens (1 mL) and mixed well. The mixture was then incubated for 15 min to lyse cells. An enzyme mixture comprising lysozyme (20 mg/mL), mutanolysin (300 U/mL), lysostaphin (40 U/mL), and lyticase (1000 U/mL) was added to the cell lysate, and then the cell lysate was incubated at 37 °C for 30 min. All enzymes are from Sigma-Aldrich.

TNA, including both DNA and RNA, was extracted. Five available commercial TNA extraction kits were tested for extraction efficiency. These kits included the BiOstic bacteremia DNA isolation kit (MOBIO, Carlsbad, CA, USA), QIAamp DNA Blood Mini Kit (Qiagen, Hilden, Germany), QIAamp UCP Pathogen Mini Kit (Qiagen, Hilden, Germany), TIANamp Blood DNA Kit (Tiangen, Beijing, China) and QIAamp cador Pathogen Mini Kit (Qiagen, Hilden, Germany). The benzyl alcohol-guanidine hydrochloride (Sigma-Aldrich, Saint Louis, USA) method was also tested^29^. The manufacturer’s instructions from the kits were followed. A total of 80 μL elution buffer was added to the TNA extraction column to elute TNA. The extracted TNA was stored at 4 °C for immediate use or −70 °C for long-term storage. Before TNA was used as the template in TAC, blue dextran 2000 (GE Healthcare, Milwaukee, WI, USA) was added at a concentration of 0.1%. A blank sample (distilled water) was included for each extraction to monitor possible contamination during TNA extraction. If the blank sample showed positive results, the entire TNA extraction batch was discarded.

### TAC performance estimation

The specificity, sensitivity, efficiency and reproducibility of the primers and probes were assessed using TNA isolated from mock (spiked) whole blood specimens. Of the 183 whole blood specimens, which were analyzed by bacterial culture tests, 102 were positive for the bacterial culture tests, and 81 were negative. Of the 151 blood culture samples, 129 were positive for the targeted pathogens on the TAC, and 22 were culture test negative. Both the whole blood specimens and the blood culture samples were used to evaluate the TAC performance. The sensitivity and specificity of the TAC assay for the targeted pathogens were determined. For blood culture samples and whole blood samples, according to the preliminary results, the cutoff Ct value was determined as 30 and 38, respectively.

### TAC reaction

TAC reaction systems followed the instructions from the SuperScript^TM^ III Platinum^TM^ One-Step qRT-PCR kit (Invitrogen, Carlsbad, CA, USA). Forty-six microliter TNA extracts containing 0.1% blue dextran 2000 were mixed with 50 μL 2 × Reaction mix, 2 μL of Superscript III platinum Taq Mix, and 2 μL of ROX reference dye to a final volume of 100 μL. The primer and the probe were placed on the TAC in advance, and the TAC was manufactured by Life Technologies. The working concentrations for primers and probes in the TAC assay were 900 nM and 250 nM, respectively. After thorough mixing, the reaction mixture was loaded into each port of the card, and the card was centrifuged at 1200 rpm for 1 min once or twice to distribute the fluid in the reaction wells. Then the card was sealed before being placed in the ViiA ^TM^ 7 real-time PCR system (Applied Biosystems, CA, USA)^11^. The cycling conditions were as follows: 50 °C for 15 min, 95 °C for 2 min, and 40 cycles of 95 °C for 15 sec and 60 °C for 45 sec. For TAC without RT, TaqMan^TM^ Universal Master Mix II (Invitrogen, Carlsbad, CA, USA) was used instead. The qPCR assay followed the instructions from the SuperScript^TM^ III Platinum^TM^ One-Step qRT-PCR kit, and the total volume was 25 μL. The reaction conditions were the same as those of the TAC above. All of the experiments were repeated to confirm the results. A negative control (one for TAC and three for qPCR) was included in each reaction.

### Statistical analysis

Data are presented as the mean ± standard deviation. The statistical analysis software SPSS 19.0 was used to analyze the data. One-way ANOVA or Student’s *t*-test was performed to compare multiple groups or 2 groups, respectively. A 2-sided *P* < 0.05 was considered statistically significant.

## Data Availability

All data included in this study are available upon request by contact with the corresponding author.
